# Development of Collagen/Demineralized Bone Powder Scaffolds and Periosteum-Derived Cells for Bone Tissue Engineering Application

**DOI:** 10.3390/ijms14012056

**Published:** 2013-01-21

**Authors:** Thakoon Thitiset, Siriporn Damrongsakkul, Tanom Bunaprasert, Wilairat Leeanansaksiri, Sittisak Honsawek

**Affiliations:** 1 Interdisciplinary Program in Biomedical Engineering, Faculty of Engineering, Chulalongkorn University, Bangkok 10330, Thailand; E-Mail: thakoonthitiset@hotmail.com; 2 Department of Chemical Engineering, Faculty of Engineering, Chulalongkorn University, Bangkok 10330, Thailand; E-Mail: siriporn.d@chula.ac.th; 3 Division of Facial Plastic and Reconstructive Surgery, Department of Otolaryngology, Faculty of Medicine, Chulalongkorn University, Bangkok 10330, Thailand; E-Mail: drtanom@yahoo.com; 4 Stem Cell Therapy and Transplantation Research Group, Institute of Sciences, School of Microbiology, Suranaree University of Technology, Nakhon Ratchasima 30000, Thailand; E-Mail: wilairat@g.sut.ac.th; 5 Department of Biochemistry, Faculty of Medicine, Chulalongkorn University, Bangkok 10330, Thailand

**Keywords:** collagen, demineralized bone powder, osteogenic differentiation, periosteum-derived cells, scaffold

## Abstract

The aim of this study was to investigate physical and biological properties of collagen (COL) and demineralized bone powder (DBP) scaffolds for bone tissue engineering. DBP was prepared and divided into three groups, based on various particle sizes: 75–125 μm, 125–250 μm, and 250–500 μm. DBP was homogeneously mixed with type I collagen and three-dimensional scaffolds were constructed, applying chemical crosslinking and lyophilization. Upon culture with human periosteum-derived cells (PD cells), osteogenic differentiation of PD cells was investigated using alkaline phosphatase (ALP) activity and calcium assay kits. The physical properties of the COL/DBP scaffolds were obviously different from COL scaffolds, irrespective of the size of DBP. In addition, PD cells cultured with COL scaffolds showed significantly higher cell adhesion and proliferation than those with COL/DBP scaffolds. In contrast, COL/DBP scaffolds exhibited greater osteoinductive potential than COL scaffolds. The PD cells with COL/DBP scaffolds possessed higher ALP activity than those with COL scaffolds. PD cells cultured with COL/DBP scaffolds with 250–500 μm particle size yielded the maximum calcium deposition. In conclusion, PD cells cultured on the scaffolds could exhibit osteoinductive potential. The composite scaffold of COL/DBP with 250–500 μm particle size could be considered a potential bone tissue engineering implant.

## 1. Introduction

Demineralized bone powder (DBP) is a well-known osteoinductive biomaterial variously used for enhancing new bone formation in orthopaedic and dental applications [1]. Several studies have shown that DBP comprises osteoinductive factors including bone morphogenetic proteins (BMPs) and transforming growth factor-β (TGF-β), that are substantial regulators for endochondral bone formation [2–4]. It has been demonstrated that BMPs extracted from DBP, implanted into small animals at heterotopic sites, could promote new bone formation [5]. DBP can be prepared by acid extraction of allograft bone, leading to loss of some calcium and mineralized components, but retains collagen and non-collagenous proteins including cytokines and growth factors [6].

Collagen (COL) is a natural biopolymer with a very large molecule comprised of repeating subunits. It is one of the longest proteins in the mammalian body and provides good biocompatibility and low antigenicity [7]. In addition, collagen responds well to cell attachment and proliferation. The combination of DBP and collagen may provide an excellent structure for tissue forming cells. The composite scaffolds of collagen and demineralized bone powder have been expected to be favorable biomaterials for bone tissue engineering.

Our previous study has shown that DBP could activate human mesenchymal stem cells to differentiate into osteoblasts by exhibiting highly expressed alkaline phosphatase activity [8]. Moreover, collagen is a good biopolymer for providing bone scaffolds. Mizuno and coworkers has illustrated that three-dimensional composite scaffolds could be designed to duplicate the packing density of *in vivo* DBP implants [9]. They have shown that DBP packed between two layers of a porous collagen assisted in the differentiation of human dermal fibroblasts into chondrocytes. Fibroblasts seeded onto the scaffolds migrated through the collagen into the packet of DBP and deposited extracellular matrix amongst the particles of DBP.

However, the effects of various particle sizes of DBP onto the physical and biological properties of the COL/DBP composite scaffolds have, as yet, not been investigated. Therefore, the purpose of this study was to develop three-dimensional osteoinductive scaffolds from demineralized bone powder and collagen composite. Moreover, the effects of various particle sizes of DBP and their influence on the physical and biological properties in combination with collagen scaffolds were evaluated. The optimum ratio of COL and DBP mixtures was determined in order to modulate their osteoinductive potential as composite scaffolds for bone tissue engineering.

## 2. Results and Discussion

### 2.1. Characteristics of Collagen/Demineralized Bone Powder Scaffolds

**Figure 1:**
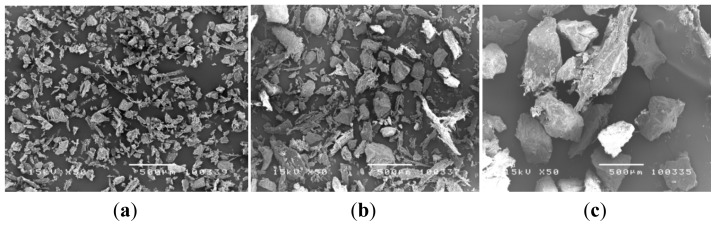
The particle size of DBP: (**a**) 75–125 μm; (**b**) 125–250 μm; (**c**) 250–500 μm. (white bar = 500 μm, magnification 50×).

**Figure 2:**
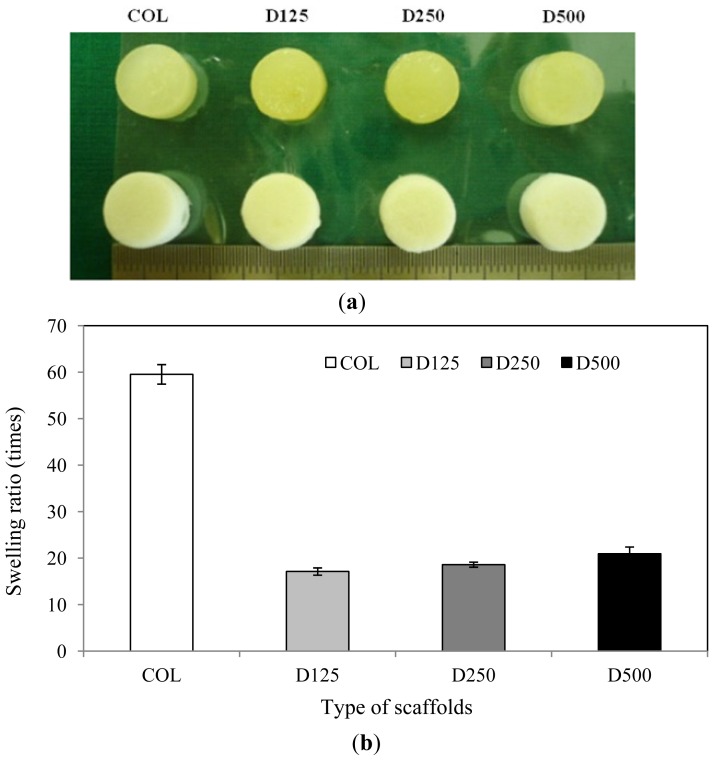
Swelling ability of the fabricated scaffolds: (**a**) Appearance of the scaffolds in wet condition and dry condition; (**b**) Average swelling ratio of the scaffolds. (*n* = 5).

**Figure 3:**
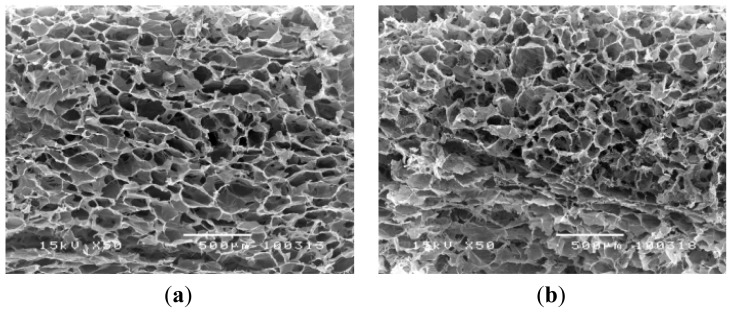

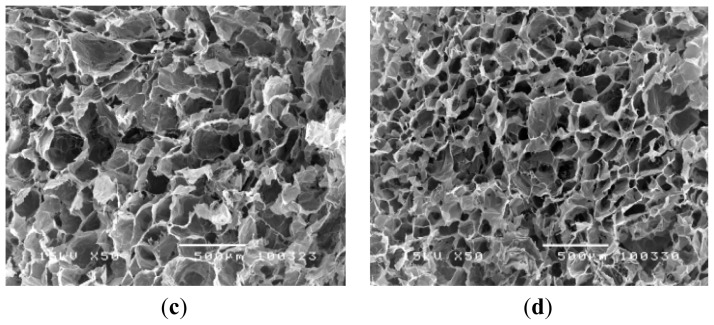
SEM micrographs of vertical cross-sections of fabricated scaffolds: (**a**) COL; (**b**) D125; (**c**) D250; (**d**) D500. (White bar = 500 μm, magnification 50×).

The particle size of DBP was characterized through SEM micrograph (Figure 1). The particle sizes of DBP were categorized into three groups as follows: 106.72 ± 22.82 μm, 199.73 ± 34.23 μm, and 354.58 ± 69.25 μm, respectively. The DBP showed random polygonal spindle shapes and clearly demonstrated different particle sizes for each group. The fabricated scaffolds have a cylindrical round shape and smooth consistency. The swelling did not affect their shape, indicating that they can stabilize their shape when soaked in phosphate-buffered saline for 24 h (Figure 2). Morphology of COL/DBP scaffolds was visualized through SEM, as shown in Figure 3. The pore sizes of all scaffolds were analyzed with the Image J program. The average pore size of the scaffolds was approximately 150 μm. In comparison with pure collagen scaffolds, the pore size of COL/DBP scaffolds was relatively smaller than that of pure collagen scaffolds.

**Figure 4:**
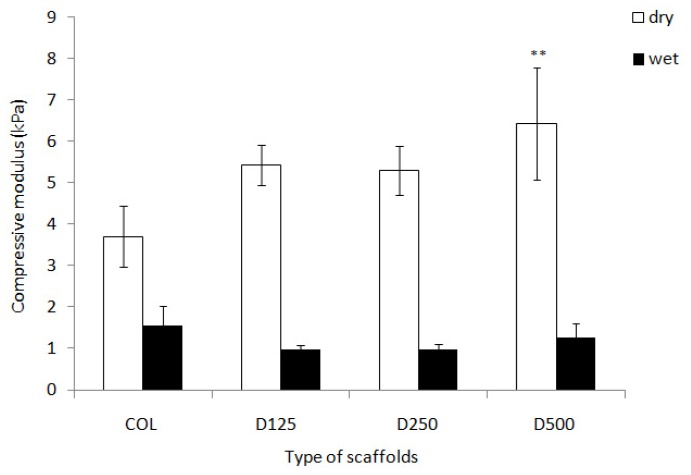
Compressive modulus and strength of the fabricated scaffolds in dry and wet conditions. Error bar represent means ± SD (*n* = 5) (*p* < 0.05).

The compression modulus data of collagen scaffolds with, and without, DBP are illustrated in Figure 4. The compression modulus of COL/DBP scaffolds with various particle sizes was higher than that of pure COL scaffolds. Yet, in wet conditions, the compression modulus of all scaffolds was decreased. However, the difference between the compression modulus of COL scaffolds and COL/DBP scaffolds was not statistically significant.

**Figure 5:**
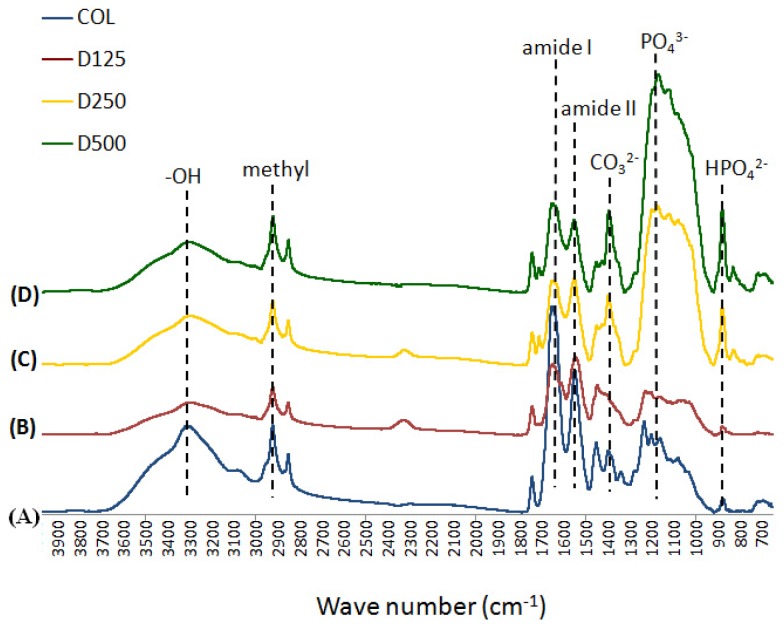
FT-IR spectra of the fabricated scaffolds: (**A**) COL; (**B**) D125; (**C**) D250; (**D**) D500.

The scaffolds were analyzed for their chemical composition by using FT-IR analysis. The FT-IR spectra obtained from type I collagen are shown in Figure 5. In the spectrum of pure collagen, four characteristic absorption bands at the frequencies of 3300, 2925, 1660, and 1550 cm^−1^ were observed. Generally, amide I bands (1660 cm^−1^) originate from C=O stretching vibrations coupled to N–H bending vibration. The amide II bands (1550 cm^−1^) arise from the N-H bending vibrations, coupled to C–N stretching vibrations. The other two bands result from the stretching vibrations of a free N–H group appearing at 2925 cm^−1^ and the vibration of the hydroxyl group, –OH, appearing at 3300 cm^−1^.

In contrast, the FT-IR spectra of COL/DBP scaffolds at various particle sizes revealed the presence of PO_4_^3−^ at the great intensity of the 1145 cm^−1^ band. HPO_4_^2−^ at 875 cm^−1^ indicates high levels of hydrogen phosphate ions. CO_3_^2−^ at 1451 cm^−1^ demonstrates the carbonyl group. These functional groups are the major components of carbonated hydroxyapatite in natural bone extracellular matrix.

### 2.2. Cell Attachment and Proliferation of PD Cells Cultured on Scaffolds

**Figure 6:**
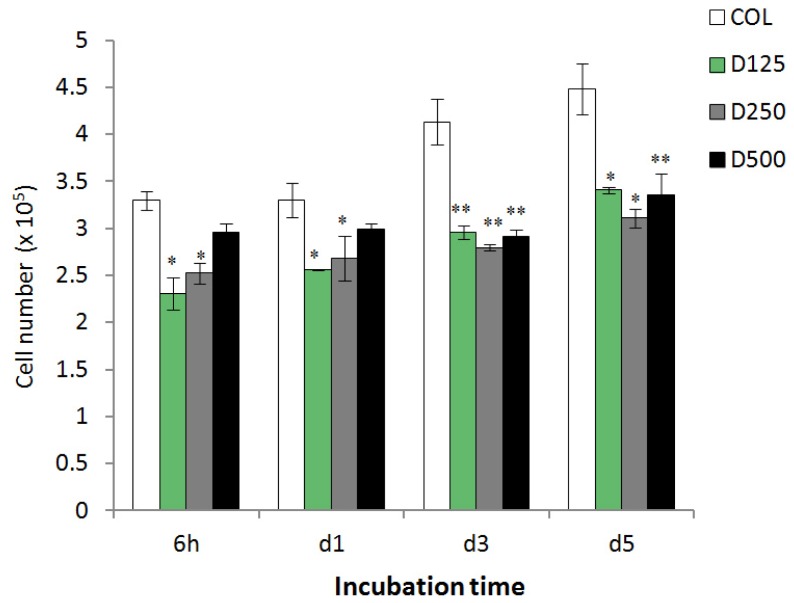
Number of PD cells proliferated on fabricated scaffolds. Error bars represent means ± SD (*n* = 3) (* *p* < 0.05 and ** *p* < 0.01).

PD cells were seeded and cultured on collagen based scaffolds impregnated with DBP of various sizes for 6 h, 1, 3, and 5 days. The numbers of attached and proliferated cells on scaffolds, analyzed by DNA assay are illustrated in Figure 6. It was observed that PD cells tended to attach and proliferate well on all scaffolds. It was also noticed that the number of PD cells proliferating on collagen blended DBP was significantly lower than on a pure collagen scaffold, indicating the hydrogel and biocompatibility effects of collagen, which promote more cell attachment and proliferation than collagen blended DBP scaffolds. In contrast, the numbers of PD cells attached and proliferating on COL/DBP scaffolds were not different. They may have an impact on osteogenic differentiation.

### 2.3. Osteoblast Differentiation of PD Cells Cultured on Scaffolds

**Figure 7:**
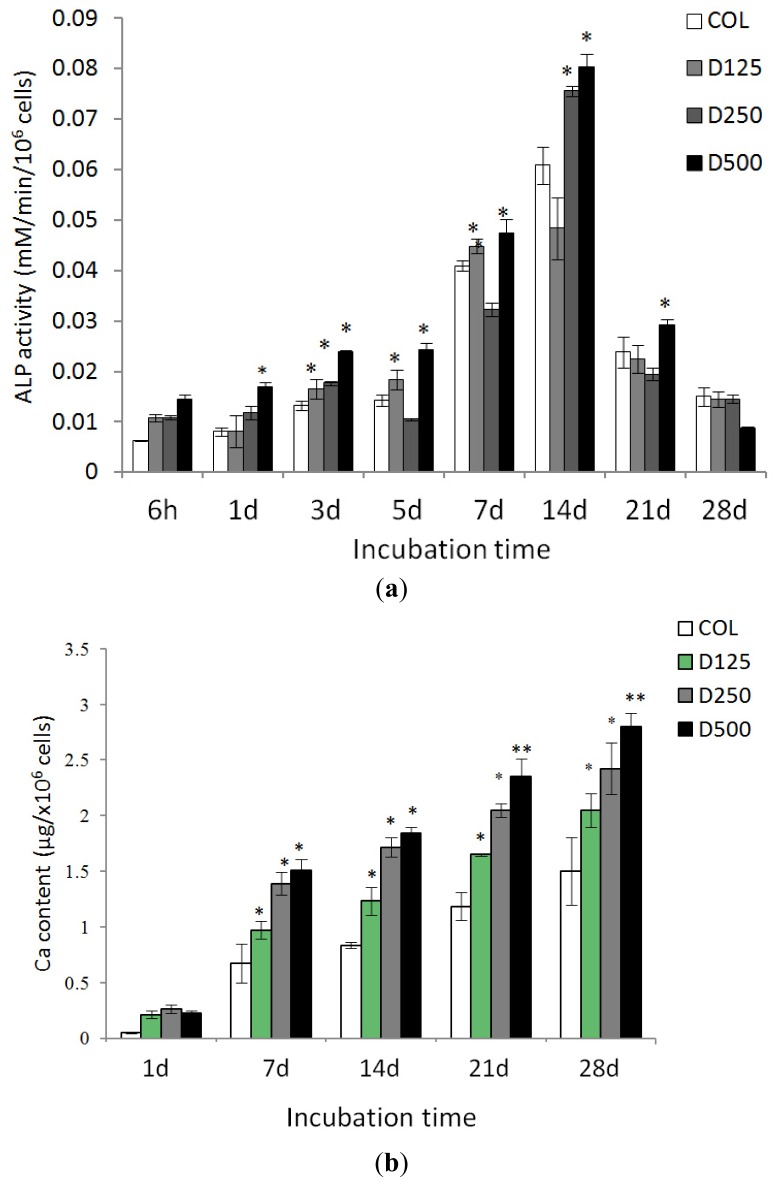
Osteogenic differentiation assay: (**a**) ALP activity of PD cells cultured on fabricated scaffolds at different times (* *p* < 0.05); (**b**) Calcium deposition of PD cells cultured on fabricated scaffolds at different times (* *p* < 0.05, ** *p* < 0.01).

ALP activities of PD cells cultured on scaffolds in osteogenic medium at various times are demonstrated in Figure 7a. ALP activities of PD cells cultured on COL blended DBP scaffolds were significantly higher than on pure collagen scaffolds. In addition, it could also be noticed that ALP activity was highest on COL scaffolds blended with DBP of 500 μm.

After 28 days, PD cells cultured on different scaffolds under osteogenic medium released increased amounts of calcium. Similar to the results of ALP activity, the amounts of calcium released from PD cells cultured on COL/DBP scaffolds were significantly higher than those on COL scaffolds (Figure 7b).

### 2.4. Morphology Analysis of PD Cells Cultured on Scaffolds

**Figure 8:**
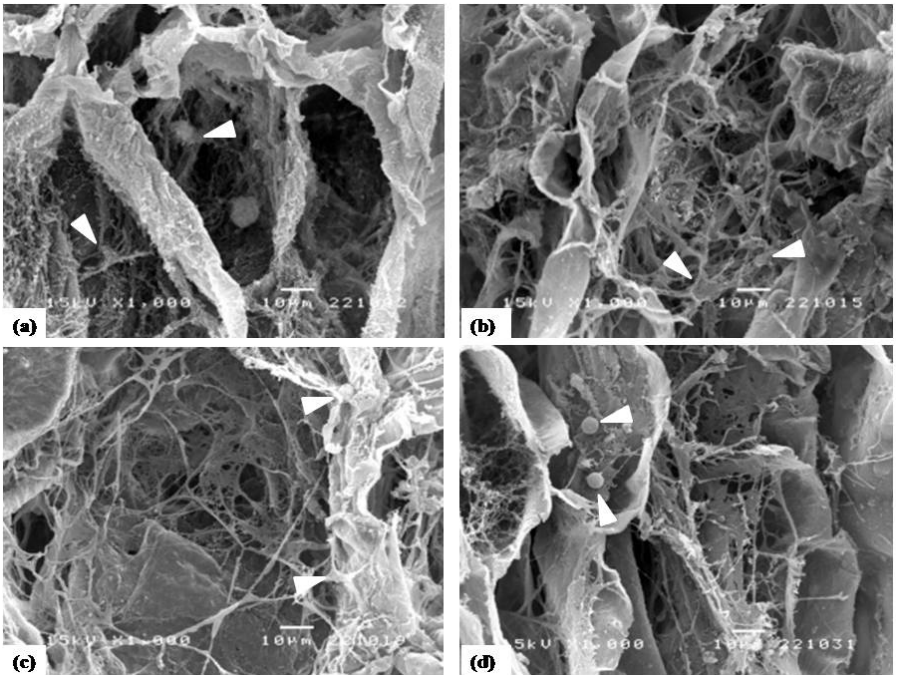
Morphology of cross-section of COL scaffold and COL/DBP scaffolds cultured with PD cells under osteogenic medium for 28 days. (**a**) COL; (**b**) D125; (**c**) D250; (**d**) D500. (scale bar = 10 μm, magnification 1000×). Human PD cells are designated by arrow heads.

The morphology of PD cells cultured on collagen-based scaffolds incorporated with various types of DBP in osteogenic medium for 28 days is illustrated in Figure 8. The SEM micrographs showed that, after 28 days of culture, PD cells formed multi-layers and the extracellular matrix (ECM) of cells was observed on all scaffolds. The scaffolds incorporated with DBP seemed to have more ECM on the surface of scaffolds. The morphology of PD cells on collagen based scaffolds incorporated with DBP implied that all scaffolds qualitatively supported the osteogenic differentiation of PD cells.

### 2.5. Chemical Composition of PD Cells Cultured with Scaffolds

**Figure 9:**
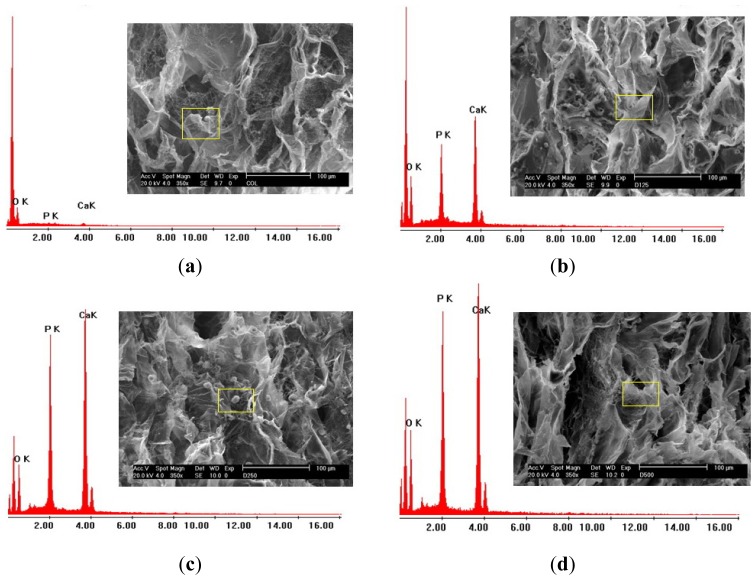
EDX analysis of fabricated scaffolds cultured with PD cells for 28 days. (**a**) COL; (**b**) D125; (**c**) D250; (**d**) D500. (scale bar = 10 μm, magnification 1000×).

After 28 days of culture, all scaffolds were analyzed by energy dispersive X-ray spectrometry (EDX) to confirm the production of calcium from PD cells. EDX analysis confirmed the above results. Calcium and phosphate contents on the cell surface were found in all scaffolds (Table 1). With the pure collagen scaffold, the calcium deposited comprised approximately 9.38%. With the scaffold containing DBP, the highest amount of calcium deposited, 43.39%, was found with 250–500 μm DBP incorporated, 35.37% with 125–250 μm DBP incorporated, and 29.81% with 75–125 μm DBP incorporated, respectively. The incorporation of DBP into collagen scaffolds engendered deposits of higher calcium concentrations compared to the pure collagen scaffolds. The EDX results corresponded to the calcium content reported in Figure 9. Our findings reveal that the main elements of the mineral are oxygen, calcium, and phosphorus.

### 2.6. Discussion

Demineralized bone powder is a promising bone graft mainly composed of collagen and bone morphogenetic proteins (BMPs) and has been widely used for bone regeneration since the 1960s [5]. DBP is considered to be the most potent osteoinductive bone particle, as it possesses the maximum surface area for interaction with target cells at the graft site [10]. Moreover, DBP provides other growth factors such as transforming growth factor-β, insulin-like growth factors, fibroblast growth factors, and platelet derived growth factors [11]. In general, TGF-β is produced and secreted by bone cells and is stored in bone matrix [12]. TGF-β is responsible for the regulation of bone turnover [13] and plays a significant role in competence for early stages of chondrogenic and osteogenic differentiation *in vitro* and *in vivo* [14]. Since TGF-β and BMPs are major osteoinductive factors in bone matrix, it is likely that osteoinduction and chondroinduction of DBP involves in TGF-β/BMP signaling pathways [12].

Tissue engineering renders new strategies to enhance the utility of biomaterials for the development of new composite scaffolds in bone regeneration. The incorporation of an osteoprogenitor cell source, coupled with its *in vitro* osteogenic differentiation before implantation, can accelerate the bone formation process within tissue-engineered biomaterials. In the present study, we investigated the ability of demineralized bone scaffolds to support the attachment and subsequent *in vitro* osteogenic differentiation of human periosteum-derived cells. We first investigated the physical property of pure collagen and COL/DBP scaffolds and determined their ability to support osteogenic differentiation of human periosteum-derived cells throughout DBP scaffolds in order to optimize a system for cell delivery.

In this study, the cellularity of pure collagen and COL/DBP scaffolds was determined by measuring DNA content as DNA quantity has been shown to exhibit a direct correlation with the cell number [15]. PD cells cultured with pure collagen scaffolds showed higher cell adhesion and proliferation than those with COL/DBP scaffolds. It is possible that the pure collagen scaffolds had signal molecules supporting cell attachment and growth. The ability of composite scaffolds to promote osteogenic differentiation of human PD cells *in vitro* was demonstrated by biochemical and morphological analysis. In our study, COL/DBP scaffolds had a tendency to promote more osteogenic differentiation than pure collagen scaffolds. Alkaline phosphatase activity is an early biochemical marker of osteogenic differentiation. Biochemical analysis showed that ALP activities of PD cells cultured on any scaffolds were peak at day 14, and decreasing thereafter. The alkaline phosphatase activity of each scaffold reached the highest level on culture-day 14. The PD cells with COL/DBP scaffolds exhibited higher ALP activity than those with pure collagen scaffolds. When PD cells are induced to differentiate into the osteoblastic lineage, they undergo sequential growth, differentiation, and maturation stages. The growth stage is recognized by an increase in cell number, the differentiation stage is evident by ALP activity, and the maturation stage is marked by mineralization. Therefore, it was understood that when the cells pass over the early stage of differentiation, ALP activity would decrease while the later maturation stage would be initiated.

Calcium content of cell lysate is indicative of late stage differentiation of osteoblasts. Therefore, we would expect to observe a continual increase in calcium content over the culture period. In the current study, all composite scaffolds that were seeded with PD cells showed a consistent elevation in calcium content, with the maximum value occurring at day 28, indicating the maturation of osteoblasts. The PD cells cultured with COL/DBP scaffolds with 250–500 μm particle size yielded the highest calcium deposition. In accordance with this finding, EDX analysis showed that COL/DBP scaffolds engendered deposits of greater calcium contents in relation to the pure collagen scaffolds. We have also used SEM images to illustrate the morphology and distribution of PD cells seeded into the scaffolds. Cells seeded into COL/DBP scaffolds presented higher quantities of ECM synthesis compared to the pure collagen scaffolds.

The present study also showed that COL/DBP scaffolds had a superior microstructure and supported differentiation of osteoblasts. Therefore, it should be conceivable that collagen in combination with DBP with 250–500 μm particle size should provide a suitable environment for differentiation of osteoblasts and could be considered a potential bone tissue engineering implant.

In the study by Aubin and colleagues, biomaterials derived from demineralized bone powder were evaluated for their ability to support scaffold interactions using ALP activity, calcium deposition, and mineralization [16]. ALP has been implicated as a key indicator that increased in enzymatic activity corresponding to an increased osteoblast phenotype *in vitro* [16]. Moreover, Kasten *et al.* reported that DBP appeared to be more suitable than hydroxyapatite and β-tricalcium phosphate to support later stages of osteoblast differentiation *in vitro* [17]. Additionally, Mauney and coworkers found that mechanical stimulation promoted osteogenic differentiation of human mesenchymal stem cells (hMSCs) on partially demineralized bone scaffolds *in vitro* [18]. However, the combination of DBP with biomaterial enhanced the osteogenic differentiation of hMSCs. Zhou *et al.* suggested that DBP combined with porous collagen sponge could be cultured with hMSCs to assess DBP stimulation of osteoblast differentiation [19]. They showed that DBP combined with collagen increased ALP activity in hMSCs more profoundly than collagen alone. Finally, DBP induced hMSCs to express the osteoblast phenotype. These studies showed that the osteoinductive potential can influence the differentiation pathway of hMSCs when stimulated with DBP. These results support the potential to engineer bone *in vitro* by using hMSCs and DBP/collagen sponges. Furthermore, they investigated if DBP induced osteogenic differentiation of hMSCs in collagen scaffolds under osteogenic medium. DBP stimulated ALP activity in hMSCs after three weeks of cultivation. Moreover, DBP have up-regulated osteoblast gene expression in hMSCs [19].

A previous study by Mauney *et al.* have investigated demineralized bone (DB) derived from cattle and fully mineralized bone (FB) as to their ability to support human bone marrow stromal cell (BMSC) osteogenic differentiation *in vitro* [18]. They demonstrated that cultivation of BMSCs on DB scaffolds displayed significantly higher ALP activity than cells maintained on the FB scaffolds.

In addition, the recent study revealed that the DBP with the size of 550–710 μm provided maximum osteoinductive potential *in vitro* and *in vivo* bioassays [20]. Vail *et al.* suggested that the particle size of equine DBP had influence on osteoinduction. The muscular implantation of DBP allograft in horses, after eight weeks, showed that DBP with medium particle size (200–1000 μm) possessed higher osteoinductive activity and exhibited minimum local inflammation whereas DBP with smaller particle size (40–200 μm) had a greater local inflammatory response [21].

## 3. Experimental Section

This study was carried out in accordance with the guidelines of the Declaration of Helsinki, and approval was granted to use human periosteum. The protocol was accepted by the ethical committee of the Faculty of Medicine, Chulalongkorn University.

### 3.1. Preparation of Biomaterials

Bovine Achilles tendon type I collagen (Sigma Aldrich, St. Louis, MO, USA) was immersed in 2 M acetic acid solution at 4 °C for 24 h. Then, the solution was stirred and homogenized at room temperature. DBP was prepared from bone allograft. Briefly, the allograft bone was cleansed with 3% hydrogen peroxide solution, and 70% isopropanol solution, respectively. The washed ground bone was pulverized into small particles and the minerals were dissolved by acid extraction. We selected pulverized bone of less than 1000 μm particle size. The bone was demineralized in 0.1 M HCl solution with continuous stirring at 16 °C for eight hours. The resulting powder was washed, lyophilized, and sieved to various particle sizes: 75–125 μm (D125), 125–250 μm (D250), and 250–500 μm (D500), respectively.

### 3.2. Fabrication of Three-Dimensional Scaffolds

The scaffolds were fabricated applying the freeze drying technique. First, the 48-well plates wrapped with aluminium foil were pre-frozen at −20 °C prior to use. The blended collagen and DBP solutions were mixed at a ratio of 60/40 (COL/DBP) at a final concentration of 2% weight/volume. The solutions were crosslinked by chemical treatment with various DBP particle sizes using 0.015% (*v*/*v*) glutaraldehyde solution. The mixed solution was developed into four groups: (1) pure collagen solution (COL); (2) collagen blended with DBP size 75–125 μm (D125); (3) collagen blended with DBP size 125–250 μm (D250); and (4) collagen blended with DBP size 250–500 μm (D500). Subsequently, the blended solutions were added to the template and immediately frozen at −20 °C and −80 °C, respectively overnight. The frozen solutions were lyophilized at −55 °C for one day. Prior to cultivation, the scaffolds were sterilized using ethylene oxide.

### 3.3. Morphology Observation and Pore Diameter Measurement

Scaffold morphology was visualized through scanning electron microscopy (SEM, Joel JSM 5400). To observe the microstructure of the scaffolds, the scaffolds were cut horizontally with a razor blade. The cut scaffolds were placed on the copper mount and sputter-coated with gold (Ion sputtering device, JFC 1100) prior to SEM observation at an accelerating voltage of 12–15 kV. The pore diameter of the scaffold was determined from SEM micrographs using the Image J Launcher program (*n* = 100).

### 3.4. Mechanical Strength Analysis

Compression tests in dry and wet conditions were performed on all scaffolds using a universal testing machine (Instron 5567) at a constant compression rate of 0.5 mm/min. The compression modulus of the scaffolds (dimension: *d* = 10 mm, *h* = 3 mm) was determined from the slope of the compression stress-strain curves during a strain range of 5%–30%. The reported values represented the mean ± standard deviation (*n* = 5).

### 3.5. Swelling Ability

Scaffolds with known weight were immersed in phosphate buffered saline (PBS) for 24 h. Subsequently, the total weight of scaffolds was examined. The ratio of water absorption (*W*_sw_) by the scaffolds was evaluated using the following equation

(1) $$W_{\text{SW}}=\frac{W_{24}-W_{0}}{W_{0}}$$

where *W*_24_ represents the wet weight of the scaffolds after 24 h of incubation, while *W*_0_ is the initial weight of the scaffolds. The values were expressed as mean ± standard deviation (*n* = 5).

### 3.6. Cultivation of Periosteum-Derived cells

Periosteum tissues were collected, with written informed consent, from a human donor at King Chulalongkorn Memorial Hospital. The periosteum-derived cells (PD cells) were prepared as follows: the tissues were rinsed several times in sterile phosphate-buffered saline to remove blood components, and the tissues were dissected into small pieces (1 mm) and placed directly into 25 cm^2^ flasks (Corning, Tewksbury, MA, USA) for culture expansion in alpha-minimum essential medium (α-MEM, Hyclone, Logan, UT, USA) containing 10% fetal bovine serum (Hyclone, Logan, UT, USA) and penicillin (100 U/mL)/streptomycin (50 μg/mL) (Hyclone, Logan, UT, USA) in a 5% CO_2_ incubator at 37 °C.

PD cells were washed once in Dulbecco’s phosphate-buffered saline (Gibco, Gaithersburg, PA, USA), incubated with 0.25% trypsin-EDTA (Gibco, Gaithersburg, PA, USA) for five minutes and brought into suspension by vigorously shaking. The trypsin reaction was blocked by adding the supernatant, and followed by another washing step. Cells were usually trypsinized at more than 80% confluency. First-passage (P1) PD cells were trypsinized and replated at 5 × 10^3^ cells/cm^2^ to yield second-passage (P2) cells, trypsinized near confluency, and subsequently yielded third-passage (P3) cells for scaffold seeding. Initiation and characterization of PD cells was accomplished as previously described [22]. Flow cytometry revealed that PD cells were positive for mesenchymal adhesion cell markers (CD29, CD44, CD90, and CD105) and negative for hematopoietic markers (CD34 and CD45) and differentiated *in vitro* into osteogenic, chondrogenic, and adipogenic cells [22].

### 3.7. Initial Cell Attachment and Proliferation Assay

PD cells (5.0 × 10^5^ cells/scaffold) were seeded onto the scaffolds and cultured in proliferation medium (α-MEM supplemented with 15% FBS) at 37 °C, 5% CO_2_. The numbers of cells after cultivation for six hours, one day, three days, and five days were measured using a DNA determination assay. Briefly, the cell samples were lysed in sodium citrate-buffered saline solution (pH 7.4) containing sodium dodecylsulfate at 37 °C overnight. Subsequently, 100 μL of cell lysate was mixed with a fluorescent dye solution (Hoechst 33258 dye) in a 96-well black plate. The fluorescence intensities of the mixed solutions were spontaneously measured at the excitation and emission wavelengths of 355 and 460 nm, respectively. The standard curve between the DNA and cell number was prepared using cells of known numbers.

### 3.8. Osteogenic Differentiation of PD Cells on Scaffolds

PD cells (1.0 × 10^6^ cells/scaffold) were seeded onto the scaffolds in culture medium under continuous shaking for six hours. After seeding for one day, the medium was substituted for osteogenic medium (α-MEM supplemented with 10% FBS, 50 μg/mL L-ascorbic acid, 10 nM dexamethasone, and 10 mM β-glycerol phosphate) and changed three times a week. The experiments were performed for four weeks in osteogenic medium, and osteogenic differentiation markers including ALP activity and calcium release were determined by *p*-nitrophenyl phosphate and *O*-cresolphythalein methods, respectively. The number of cells was measured through a DNA determination assay and used for normalization of the ALP activities and calcium contents.

Alkaline phosphatase activity was measured using alkaline phosphatase activity assay (Sigma, St. Louis, MO, USA). Briefly, one milliliter aliquot samples of cell lysate were mixed with 0.2 mL 100 μmole/mL p-nitrophenyl phosphate in 0.15 M 2-amino-2-methyl-1-propanol buffer, pH 10.4, and incubated in a 37 °C water bath for 15 min. The reaction was stopped by addition of 50 μL 1.0 N NaOH and absorbance at 405 nm was measured. The alkaline phosphatase activities were expressed as units of enzyme (mM p-nitrophenyl/min/10^6^ cells).

Calcium content was then determined using the *O*-cresolphythalein complexone calcium binding assay according to the manufacturer’s instructions (DMA calcium assay, Sigma, St. Louis, MO, USA). Briefly, the calcium in cell lysate was extracted in 1.0 N HCl at 4 °C for four hours. 50 μL of sample or standard were mixed with 4.0 mL of calcium working reagent. Calcium working reagent was prepared by mixing equal amounts of calcium color reagent and calcium base reagent. Absorption was measured at 570 nm. Calcium concentrations were calculated from a standard curve using known concentrations of DMA calcium standard.

After 28 days of osteogenic differentiation, the cell seeded scaffolds were washed with PBS to remove non-adherent cells and then fixed in 2.5 wt% glutaraldehyde solution in PBS at 4 °C for one hour. Subsequently, the scaffolds were dried using the critical point dry technique. Dried scaffolds were cut into cross sections and observed under SEM.

### 3.9. Elemental Analysis of Cell Surface Cultured Scaffolds

Elements, especially calcium (Ca), phosphorous (P), and oxygen (O) on the cell surface after cultivation for 28 days in osteogenic media were analyzed by energy-dispersive X-ray spectroscopy (EDX, Philip Model XP 30 CP). The same cell seeded constructs used for SEM observation were used for EDX analysis.

### 3.10. Statistical Analysis

All assays were repeated in two independent experiments with a minimum of *n =* 3 for each data point. The results of one independent experiment are demonstrated for each analysis. All statistical analyses were performed with SPSS statistical package, version 17.0 (SPSS Inc., Chicago, IL, USA). Data were expressed as mean ± standard deviation (SD). The student’s unpaired *t*-test was used for group comparisons where appropriate. *p* < 0.05 were considered as statistically significant.

## 4. Conclusions

Upon compression testing, the COL/DBP scaffolds showed better physical properties than the COL scaffolds while their morphology did not differ. This study has established that collagen scaffolds with incorporated DBP could effectively promote attachment and proliferation of PD cells. Furthermore, the COL/DBP scaffolds could more profoundly enhance osteogenic differentiation than pure collagen scaffolds. Our findings demonstrated the higher calcium production of PD cells cultured on the COL/DBP scaffolds as compared to the COL scaffolds. Furthermore, DBP 250–500 μm in size exhibited the most pronounced effects on *in vitro* osteogenic differentiation and provided the optimal environment for bone regeneration. Future studies could focus on using fibroblasts and differentiated osteoblasts seeded onto the scaffolds for negative and positive controls, respectively.

## Figures and Tables

**Table 1:** Percentage of surface elements of periosteum-derived cells (PD cells) cultured on the scaffolds.

Type of scaffolds	O (%)	Ca (%)	P (%)	Ca/P ratio
COL	71–90	6–15	4–14	1.04–1.47
D125	15–52	29–57	19–28	1.53–2.01
D250	38–82	14–39	9–23	1.32–1.61
D500	51–63	22–31	14–18	1.58–1.74
